# Associations of *ORMDL1* gene copy number variations with growth traits in four Chinese sheep breeds

**DOI:** 10.5194/aab-62-571-2019

**Published:** 2019-10-21

**Authors:** Xiaogang Wang, Xiukai Cao, Yifan Wen, Yilei Ma, Ibrahim Elsaeid Elnour, Yongzhen Huang, Xianyong Lan, Buren Chaogetu, Linyong Hu, Hong Chen

**Affiliations:** 1 Key laboratory of Animal Genetics, Breeding and Reproduction of Shaanxi Province, College of Animal Science and Technology, Northwest A&F University, Yangling, Shaanxi 712100, China; 2 Animal Disease Control Center of Haixi Mongolian and Tibetan Autonomous Prefecture, Delingha, Qinghai 817000, China; 3 Key Laboratory of Adaptation and Evolution of Plateau Biota, Northwest Institute of Plateau Biology, Chinese Academy of Sciences, Xining, Qinghai, 810001, China

## Abstract

Copy number variations (CNVs) are gains and losses of genomic sequence of more than 50 bp between two individuals of a species. Also, CNV is considered to be one of the main elements affecting the phenotypic diversity and evolutionary adaptation of animals. ORMDL sphingolipid biosynthesis regulator 1 (*ORMDL1*) is a protein-coding gene associated with diseases and development. In our study, the polymorphism of *ORMDL1* gene copy numbers in four Chinese sheep breeds (abbreviated CK, HU, STH, and LTH) was detected. In addition, we analyzed the transcriptional expression level of *ORMDL1* gene in different tissues of sheep and examined the association of *ORMDL1* CNV with growth traits. The statistical analysis revealed that *ORMDL1* CNV was remarkably correlated with body height, heart girth, and circumference of cannon bone in HU sheep (P<0.05), and there are significant effects on body weight, body height, body length, chest depth, and height of hip cross in STH sheep (P<0.05). In conclusion, our results provide a basis for the relationship between CNV of *ORMDL1* gene and sheep growth traits, suggesting that *ORMDL1* CNV may be considered a promising marker for the molecular breeding of Chinese sheep.

## 1 Introduction

China has a rich resource of native sheep breeds (Zhao and Li, 2017), some of which possess specific traits such as fresh and tender meat, strong stress resistance, and so on. Breeding sheep with excellent growth traits by molecular breeding technology has become an essential means of breeding. Copy number variation (CNV) might be one of the main factors affecting phenotypic diversity and evolutionary adaptation in animals, employing a wide variety of mechanisms, such as gene dosage and transcript structure alterations, to modulate organismal plasticity (Clop et al., 2012). There are four kinds of the forming mechanism of CNV, consisting of non-allelic homologous recombination (NAHR), non-homologous end-joining (NHEJ), fork stalling and template switching (FoSTeS), and L1-mediated retrotransposition (Gu et al., 2008). The recent studies showed that the different copy number types of genes in the sheep genome had different effects on the growth and development of sheep, such as several critical CNV-overlapping genes (*BTG3*, *PTGS1*, and *PSPH*) which were involved in fetal muscle development, prostaglandin (PG) synthesis, and bone color (Yang et al., 2018a). According to reports, the CNV map of the Chinese native sheep genome has been successfully constructed based on the Illumina *Ovine* SNP 600 K BeadChip array (Ma et al., 2017). Moreover, CNV detection and map construction have been accomplished in chicken (Wang et al., 2010), goat (Fontanesi et al., 2010), and cattle (Bae et al., 2010; Fadista et al., 2010).

ORMDL families consist of three genes: *ORMDL1*, *ORMDL2*, *ORMDL3*. ORMDL sphingolipid biosynthesis regulator 1 (*ORMDL1*) is a protein-coding gene which is related to sphingolipid metabolism, metabolism, ceramide homeostasis, and disease. An important paralog of this gene is *ORMDL2*. Studies have shown that ORMDL proteins are the primary regulators of ceramide biosynthesis and mediate feedback regulation of ceramide biosynthesis in mammalian cells (Siow and Wattenberg, 2012). ORMDLs may be involved in the regulation of ceramides during IL-1-mediated sterile inflammation (Cai et al., 2016). Studies have confirmed that polymorphisms in *ORMDL* genes are associated with asthma; increased ORMDL levels in asthmatic patients indicate that ORMDLs can cause asthma (Toncheva et al., 2015). In recent years, the ORMDL protein is a response to excess cholesterol, leaving the endoplasmic reticulum (ER) to activate serine palmitoyl-CoA transferase (SPT) and increase sphingomyelin biosynthesis, which buffers excess cellular cholesterol (Wang et al., 2015). Besides, the fact that human ORMDL homologs can rescue yeast mutants indicated that ORMDL is involved in protein folding in the ER (Hjelmqvist et al., 2002). We hypothesize that it may improve the function of *ORMDL1* gene and affect growth traits by regulating metabolism and ceramide levels in sheep.

In this study, we found that the *ORMDL1* gene has a copy number variation region (Chr2: 118 432 001–118 434 800 bp, 2800 bp), which is closely related to growth traits in sheep. Based on the Animal QTL Database, we found that the *ORMDL1* CNV region is involved in a high density of quantitative trait loci (QTLs) for various traits of economic importance, especially muscle and fat development (Table 1). Moreover, there is no literature reported on the relationship between the copy number variation of *ORMDL1* gene and growth traits in sheep. We analyzed the associations of *ORMDL1* CNV with growth traits in four Chinese sheep breeds. The result shows that CNV of *ORMDL1* gene may be used as a molecular marker for the sheep breeding.

**Table 1:** QTLs associated with *ORMDL1* CNV.

QTL type	QTL ID	QTL region	P value
muscle-to-bone ratio QTL	13 763	117 854 679–118 644 634	<0.001
Shoulder bone weight QTL	13 764	117 854 679–118 644 634	<0.05
Shoulder fat weight QTL	13 765	117 854 679–118 644 634	<0.001
Shoulder muscle percentage QTL	13 766	117 854 679–118 644 634	<0.001
Shoulder bone percentage QTL	13 767	117 854 679–118 644 634	<0.001
Shoulder fat percentage QTL	13 768	117 854 679–118 644 634	<0.001
Myosin isoform type IIb content QTL	13 769	117 854 679–118 644 634	<0.001
Myosin isoform type IIa content QTL	13 773	117 854 679–118 644 634	<0.001
Myosin isoform type I content QTL	13 777	117 854 679–118 644 634	<0.05
Milk fat yield {180d} QTL	169 383	117 854 679–118 644 634	<0.05
Meat color L${}^{*}$ QTL	14 163	117 854 679–118 644 634	<0.05
Milk protein percentage QTL	57 738	117 854 679–118 644 634	<0.05
Loin fat thickness QTL	13 730	117 854 679–118 644 634	<0.05
Leg muscle weight QTL	13 737	117 854 679–118 644 634	<0.05
Soft tissue depth at GR site QTL	13 724	117 854 679–118 644 634	<0.05
Longissimus muscle width QTL	13 726	117 854 679–118 644 634	<0.05
Subcutaneous fat weight QTL	13 738	117 854 679–118 644 634	<0.05
Leg fat weight QTL	13 740	117 854 679–118 644 634	<0.05
Milk fat percentage QTL	13 915	117 854 679–118 644 634	<0.05

The meat color was determined using a Minolta ChromaMeter (Minolta Co., Ltd., Osaka, Japan) calibrated to a white standard using the L${}^{*}$, a${}^{*}$, b${}^{*}$ scale. Color L${}^{*}$ (lightness), color a${}^{*}$ (redness), color b${}^{*}$ (yellowness). Note that “GR” is the soft tissue depth 110 mm off the midline in the region of the 12th rib.

## 2 Materials and methods

### 2.1 Animals and growth trait measurements

All experiments were approved by the Review Committee for the Use of Animal Subjects of Northwest A&F University. Four Chinese sheep breeds were tested in this study so as to detect the intergroup distribution of *ORMDL1* gene copy number variations: Chaka sheep (CK, n=300, Haixi state, Qinghai province, China), Hu sheep (HU, n=198, Pingle town, Mengjin county, Henan province, China), Small-tailed Han sheep (STH, n=182, Yongjing county, Gansu province, China), and Large-tailed Han sheep (LTH, n=54, Yongjing county, Gansu province, China). As shown in Table S1 in the Supplement, the information for sex and age of four Chinese sheep breeds is provided. All 734 sheep were used to detect the frequency distributions of CNVs. All of the individuals were selected randomly as test cases. Moreover, the individuals from each breed were selected from the same breeding farm. Blood samples were obtained and genomic DNA was isolated from leukocytes with phenol–chloroform extraction. The phenotypic data of growth traits such as body weight, body length, body height, chest depth, heart girth, pipe circumference, rump width, and so on were recorded and collected for all adults in four breeds for the CNV association analysis.

### 2.2 Genomic DNA extraction, RNA isolation, and cDNA synthesis

All blood samples of the sheep were collected and genomic DNA was extracted from the blood samples (2 mL) by proteinase K digestion, extraction, and the phenol–chloroform methods. Tissue samples (heart, liver, spleen, lung, kidney, muscle, fat) were collected from the slaughterhouse and quickly put into liquid nitrogen. Total RNA was isolated from the different tissues with Trizol reagent (TaKaRa, Otsu, Shiga, Japan) according to the manufacturer's protocol and treated with RNase-free DNase (TaKaRa). Concentrations and purity of RNA were measured by spectrophotometry. First-strand complementary DNA (cDNA) was synthesized using a PrimeScript RT Reagent Kit (Perfect Real Time) (Clontech, TaKaRa) with 1 µg of total RNA as the template.

### 2.3 Primer design and amplification detection

**Figure 1:**
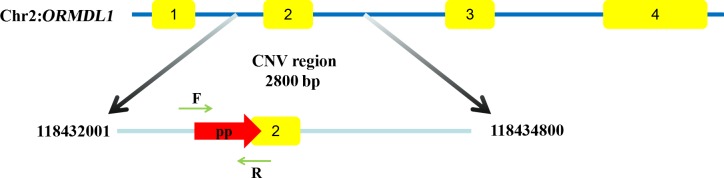
The region of *ORMDL1 *gene CNV in sheep breeds. Yellow boxes represent coding regions. The CNV region starts from 118 432 001 to 118 434 800 in Chr2 (Oar_v4.0). The pp (primer pair CNV) starts from 118 432 975 to 118 433 118 and the detection sequence size is 144 bp.

The *ORMDL1* has CNV in pig and cattle according to the Animal Omics Database (http://animal.nwsuaf.edu.cn/code/index.php/main/loadByGet?address[]=main/html/panGenomeDB.php, last access: 17 September 2019). Further validation of the CNV region, including the *ORMDL1* gene, was performed for sheep in this study. Our unpublished CNV data (i.e., Huang et al., unpublished data) about sheep indicated that the *ORMDL1* CNV region, *ORMDL1*-CNV, is located in the *ORMDL1* gene reference genome sequence (Oar_v4.0) from Chr2: 118 432 001–118 434 800 bp, a total of 2800 bp (Fig. 1). The primer was designed in the CNV region of *ORMDL1* gene; meanwhile, the reference primer was designed using NCBI Primer-BLAST. The primer information is shown in Table 2; as illustrated in Fig. 1, the amplified fragment by primer pair (*ORMDL1*-CNV) was in the CNV region.

**Table 2:** The information of primers used in this study.

	Locus	Primer sequence (5${}^{\prime }$ to 3${}^{\prime }$)	Amplification length (bp)
DNA level	*ORMDL1*-CNV	F :5${}^{\prime }$-CCCAGTAGCACACTTATTTTGTC-3${}^{\prime }$	144 bp
		R: 5${}^{\prime }$-CCGGGTATTCGGATTCACCT-3${}^{\prime }$	
	*ANKRD1*	F: 5${}^{\prime }$-TGGGCACCACGAAATTCTCA-3${}^{\prime }$	143 bp
		R: 5${}^{\prime }$-TGGCAGAAATGTGCGAACG-3${}^{\prime }$	
mRNA level	*ORMDL1*	F: 5${}^{\prime }$-GACCAGGGTAAAGCAAGGCT-3${}^{\prime }$	179 bp
		R: 5${}^{\prime }$-AGCACACTCAGGAGAGAAGC-3${}^{\prime }$	
	*GAPDH*	F: 5${}^{\prime }$-TGAGGACCAGGTTGTCTCCTGCG-3${}^{\prime }$	145 bp
		R: 5${}^{\prime }$-CACCACCCTGTTGCTGTAGCCA-3${}^{\prime }$	

### 2.4 Copy number analysis of *ORMDL1* gene

In this study, we researched the relative copy numbers of sheep *ORMDL1* gene. Ankyrin repeat domain 1 (*ANKRD1*) was chosen as the internal reference gene because there is neither CNVs nor segmental duplication in the Database of Genomic Variants of *ANKRD1*. The copy number of *ORMDL1* gene was confirmed based on the assumption that there were two copies of the DNA segment in the calibrator animals. Genomic quantitative polymerase chain reaction (qPCR) experiments were conducted using SYBR^®^ Green in triplicate reactions. A total of 12.5 µL reaction mixtures contained 10 ng of DNA, 6.25 µL 2× RealStar Green Fast Mixture (GenStar, Beijing, China), and 10 pmol of primers. Thermal cycling conditions consisted of one cycle of 10 min at 95 ${}^{\circ }$C followed by 40 cycles of 15 s at 95 ${}^{\circ }$C, 60 s at 60 ${}^{\circ }$C, and 30 s at 72 ${}^{\circ }$C.

### 2.5 Expression profiling of *ORMDL1* in sheep

The expression levels of *ORMDL1* in different tissues of sheep were detected using qPCR on a CFX 96^™^ real time detection system (Bio-Rad, Hercules, CA, USA). The gene's relative expression was normalized to the expression of the sheep *GAPDH* gene. The qPCR experiment was performed using *ORMDL1* and *GAPDH* gene-specific primer pairs (Table 2). The *ORMDL1* gene expression levels were quantified using Gene Expression Macro software (Applied Biosystems, Life Technologies, Carlsbad, CA, USA) by employing an optimized comparative Ct (ΔΔCt) value method, commonly designated as 2${}^{-\Delta \Delta \mathrm{Ct}}$. All the experiments were repeated three times and the result was presented as mean ± SD.

**Figure 2:**
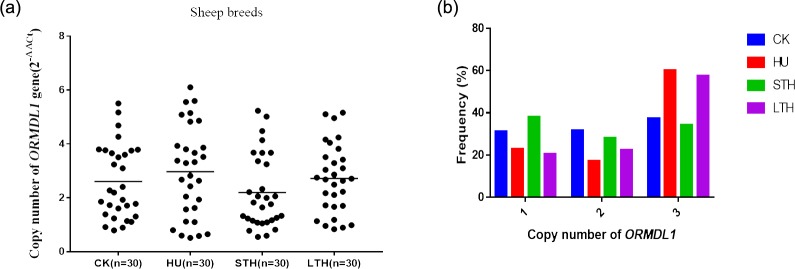
The distribution of *ORMDL1* gene copy numbers in different sheep breeds. **(a)** The distribution of *ORMDL1* gene copy number in four Chinese sheep breeds. **(b)** The proportion of subjects with relative copy numbers of the *ORMDL1* gene in the four Chinese sheep breeds.

### 2.6 Statistical analysis

In our study, the association of *ORMDL1* copy numbers and mRNA expression levels were analyzed using $2\times 2^{-\Delta \Delta \mathrm{Ct}}$ (L. Yang et al., 2017; M. Yang et al., 2017) and $2^{-\Delta \Delta \mathrm{Ct}}$ respectively. The copy number of *ORMDL1* was divided into three types: gain (copy number >2), loss (copy number <2), and median (copy number = 2). The associations of *ORMDL1* CNV with growth traits in CK, HU, STH, and LTH sheep breeds were analyzed using SPSS v18.0 software (SPSS, Inc., Chicago, IL, USA) via the general linear model method. The effects on phenotypic traits were statistically analyzed using the following model: $Y_{ijkl}=\mu +A_{i}+s_{j}+{\mathrm{CNV}}_{k}+e_{ijkl}$, where $Y_{ijkl}$ is the observation of the growth traits, μ is the overall mean of each trait, $A_{i}$ is the effect due to ith age, $S_{j}$ is the effect due to jth sex, CNV${}_{k}$ is the effect of jth CNV type of *ORMDL1* gene, and $e_{ijkl}$ is the random residual error.

## 3 Results

### 3.1 Distribution of CNVs of *ORMDL1* in four sheep breeds

In order to investigate the distribution of *ORMDL1* CNVs in different sheep breeds, we detected the copy number of *ORMDL1* in CK, HU, STH, and LTH breeds. The CNV types were divided into gain (copy number >2), loss (copy number <2), and median (copy number = 2) based on the $2\times 2^{-\Delta \Delta \mathrm{Ct}}$ values. First, we selected 30 of each sheep breed to study whether the *ORMDL1* gene has CNV. As is shown in Fig. 2a, the distribution of *ORMDL1* CNV shows that the gain of copy number was more dominant than loss and median in CK, HU and LTH sheep, while the copy number loss was more dominant than gain and median in STH sheep. In addition, Fig. 2b displays the proportion of subjects with relative copy numbers of the *ORMDL1* gene in the four breeds. It can be seen that the frequency of sheep (CK, HU, LTH) with a copy number of >2 was much higher than sheep with other copy numbers, especially in the HU breed (60 %). The copy numbers main varying from one copy to three copies was identified in the four breeds. All breeds show varying types of gain and loss, suggesting that the diversity of variation appeared in the *ORMDL1* gene CNV region.

**Figure 3:**
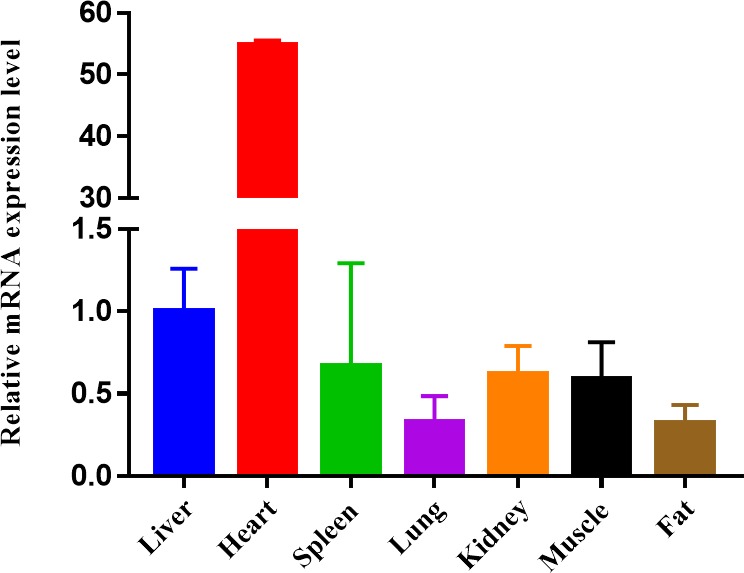
Expression profiling of *ORMDL1* gene in different tissues in sheep. The values are the averages of three independent experiments measured by $2^{-\Delta \Delta \mathrm{Ct}}$. Error bars represent the standard deviation (SD) (n=3), the relative mRNA expression levels of *ORMDL1* gene are normalized, and *GAPDH* was used as internal reference.

**Table 3:** Statistical association analysis of sheep *ORMDL1* gene copy number variations with growth traits in CK sheep.

Growth traits	CNV types (mean ± SE)			P value
	Gain (n=112)	Loss (n=93)	Median (n=95)	
Body weight (kg)	58.460±1.534	57.173±1.530	57.986±1.390	0.721
Body height (cm)	67.615±0.510	67.694±0.509	67.596±0.462	0.982
Body length (cm)	74.987±0.848	74.193±0.845	74.246±0.768	0.601
Heart girth (cm)	90.777±1.026	90.648±1.024	91.044±0.930	0.938

**Table 4:** Statistical association analysis of sheep *ORMDL1* gene copy number variations with growth traits in HU sheep.

Growth traits	CNV types (mean ± SE)			P value
	Gain (n=119)	Loss (n=45)	Median (n=34)	
Body weight (kg)	31.882±0.418	33.205±0.687	32.812±0.782	0.209
Body height (cm)	$61.466^{A}\pm 0.325$	$63.700^{B}\pm 0.529$	$60.676^{A}\pm 0.608$	0.00024${}^{**}$
Body length (cm)	70.756±0.351	70.700±0.571	71.879±0.667	0.299
Rump width (cm)	17.561±0.105	17.096±0.170	17.471±0.196	0.068
Heart girth (cm)	$76.017^{a}\pm 0.408$	$77.989^{b}\pm 0.664$	$77.824^{b}\pm 0.764$	0.014*
Circumference of cannon bone (cm)	$7.076^{a}\pm 0.049$	$7.295^{b}\pm 0.081$	$6.985^{a}\pm 0.092$	0.025${}^{*}$

Values with different superscripts (e.g., a, b) within the same row differ significantly at ${}^{*}$ P<0.05. Values with different superscripts (e.g., A, B) within the same row differ significantly at ${}^{**}$ P<0.01.

### 3.2 Association analysis between *ORMDL1* CNV and growth traits in sheep breeds

In recent years, many studies have reported that CNVs are associated with human height and livestock growth as well as growth traits (Kim et al., 2013; Zhang et al., 2018). In this study, association analysis of *ORMDL1* CNVs with growth traits was performed in four sheep breeds (CK, HU, STH, LTH) by a general linear model. As is shown in Tables 3–6, no significant differences were detected among the *ORMDL1* CNV types with growth traits in CK and LTH sheep (P>0.05) but there was a tendency that individuals with copy number gain had better traits than those with copy number loss or median in CK sheep. However, in the HU sheep population, there are significant effects on body height, heart girth, and circumference of cannon bone (P<0.05), especially with the body height (P=0.00024), and the individuals with copy number loss had higher values than those with gain or median; Similarly, the type of gain has better growth traits than loss and median in body weight, body height, body length, chest depth, and height of hip cross (P<0.05), especially with the body weight (P=0.001), body height (P=0.009), and height of hip cross (P=0.00). These results show that the CNV of *ORMDL1* gene has a remarkable influence on sheep's growth traits (P<0.05).

**Table 5:** Statistical association analysis of sheep *ORMDL1* gene copy number variations with growth traits in STH sheep.

Growth traits	CNV types (mean ± SE)			P value
	Gain (n=62)	Loss (n=69)	Median (n=51)	
Body height (cm)	$64.754^{A}\pm 0.528$	$61.964^{B}\pm 0.500$	$63.789^{A}\pm 0.608$	0.001${}^{**}$
Body length (cm)	$60.821^{A}\pm 0.793$	$57.525^{B}\pm 0.751$	$59.748^{A}\pm 0.913$	0.009${}^{**}$
Heart girth (cm)	$73.274^{a}\pm 0.765$	$70.496^{b}\pm 0.725$	$72.647^{\mathrm{ab}}\pm 0.881$	0.023${}^{*}$
Circumference of cannon bone (cm)	7.259±0.085	7.185±0.081	6.997±0.098	0.117
Chest breadth (cm)	18.634±0.426	19.493±0.404	19.807±0.491	0.152
Chest depth (cm)	$27.665^{\mathrm{ab}}\pm 0.332$	$27.039^{a}\pm 0.315$	$28.304^{b}\pm 0.388$	0.038${}^{*}$
Height of hip cross (cm)	$65.333^{A}\pm 0.507$	$61.443^{B}\pm 0.480$	$62.530^{B}\pm 0.584$	0.000${}^{**}$

Values with different superscripts (e.g., a, b) within the same row differ significantly at ${}^{*}$ P<0.05. Values with different superscripts (e.g., A, B) within the same row differ significantly at ${}^{**}$ P<0.01.

**Table 6:** Statistical association analysis of sheep *ORMDL1* gene copy number variations with growth traits in LTH sheep.

Growth traits	CNV types (mean ± SE)			P value
	Gain (n=31)	Loss (n=11)	Median (n=12)	
Body weight (kg)	58.550±5.511	53.548±6.116	59.914±6.116	0.781
Body height (cm)	76.228±2.292	78.371±2.378	76.277±2.588	0.808
Body length (cm)	75.883±2.443	77.233±2.535	77.505±2.759	0.873
Circumference of cannon bone (cm)	7.520±0.306	7.940±0.317	7.611±0.345	0.668
Chest breadth (cm)	22.054±1.169	24.048±1.213	23.074±1.320	0.537
Heart girth (cm)	91.795±3.726	99.002±3.866	96.372±4.209	0.424
Height of hip cross (cm)	93.372±6.006	84.571±7.102	76.834±6.215	0.210

### 3.3 The mRNA expression level of the *ORMDL1* gene in sheep tissues

To study the potential mechanism of action of CNV, here we show the mRNA expression profiling of *ORMDL1* in seven different tissues (liver, heart, spleen, lung, kidney, muscle, fat) of sheep (Fig. 3). Obviously, the mRNA of *ORMDL1* is widely expressed in the tissues to be tested, with the highest expression level in the heart and liver, a medium expression level in the spleen, kidney, and muscle; and relatively low expression levels in lung and fat.

## 4 Discussion

Genomic polymorphisms exist in various forms including single-nucleotide variations, translocations, insertions, and CNVs (Kalman and Vitale, 2009; McKnight et al., 2010; Yang et al., 2016). Due to the broader coverage of CNV and the more considerable variation in genomic structure, more and more research on CNV has been conducted in recent years. Copy number variations have been found in human and livestock genomes with the development of whole-genome sequencing, and CNV is mainly used to study human diseases (Henrichsen et al., 2009) such as neurodevelopmental disorders (Takumi and Tamada, 2018). In animal breeding, according to reports, copy number variations can affect the economic traits of animals and lay the foundation for molecular breeding, such as in pig (Fowler et al., 2013), chicken (Lin et al., 2018), sheep (Ma et al., 2017), and cattle (Xu et al., 2014; Zheng et al., 2019). Studies have shown that a comprehensive sheep CNV map has been generated, equivalent to 6.9 % of the sheep genome (Yang et al., 2018b). However, it is necessary to use a large number of experimental individuals from various sheep breeds to identify the existence of gene CNVs and to study whether specific CNVs affect individuals. Therefore, in this study, we reported for the first time that the CNVs of the *ORMDL1* gene were significantly associated with certain growth traits in the HU and STH sheep breeds.

There are many methods for the detection of copy number variation, such as SNP chip, comparative genomic hybridization (CGH) (Pinto et al., 2011), qPCR assay, and next-generation sequencing (NGS). However, qPCR is widely used as a promising means of verifying CNV due to its simple and practical qPCR assay (Xu et al., 2013). In our study, the ΔΔCt analysis and the reference gene *ANKRD1* were used to confirm the selected CNV region and also to first analyze the *ORMDL1* gene mRNA expression levels in different tissues of sheep. These results show that the expression level of *ORMDL1* in heart and liver is higher than other tissues of sheep, suggesting that the *ORMDL1* gene might play a critical role in heart development. The liver is the main organ of metabolism; therefore, we think that the effect on growth traits may be achieved through adaptive means. *ORMDL1* is an important gene involved in sphingolipid metabolism, so we speculate that the *ORMDL1* gene influences the sphingolipid metabolism in the liver. In addition, the *ORMDL1* gene might also affect the development of muscle in sheep.

There is a difference in the distribution of *ORMDL1* copy number among the four sheep breeds; this may be attributed to the diverse genetic background among breeds (Lehnert et al., 2007) or may be affected by the growing environment. The CNV region has been detected in many functional genes, and the copy number may influence the transcriptional and phenotypic variations through the dose effect. Some studies have reported that CNVs have potential effects on economic and reproductive traits in livestock (Hou et al., 2011). Additionally, some QTLs spanning the CNV fragments were related to chicken body weight, carcass weight, and breast muscle mass (Wang et al., 2012). In our study, the results show that the *ORMDL1* gene significantly associated with many growth traits in HU and STH sheep, especially with body weight, body height, and heart girth. This may be due to the dose effect of the *ORMDL1* gene, which in turn affects the development and deposition of muscles, leading to significant differences in body weight and heart girth.

## 5 Conclusion

In short, we detected and validated the copy number variations in the *ORMDL1* gene in different Chinese sheep breeds. In addition, we have examined the expression of *ORMDL1* gene in various tissues of sheep at the mRNA level. Correlation analysis shows that the *ORMDL1* gene CNV had significant effects on the growth traits of Chinese sheep, especially in the HU and STH sheep. Therefore, our study provides elementary evidence for the functional roles of the *ORMDL1* CNV in larger populations and different sheep breeds, which may provide a new idea for the potential application of CNV in sheep breeding as new molecular markers in the future.

## Supplement

10.5194/aab-62-571-2019-supplement The supplement related to this article is available online at: https://doi.org/10.5194/aab-62-571-2019-supplement.

## Data Availability

The original data are available upon request to the corresponding author.
