# Supplementary material for: Associations of ORMDL1 gene copy number variations with growth traits in four Chinese sheep breeds
Source: Arch Anim Breed. 2019 Oct 21;62(2):571–8. doi: 10.5194/aab-62-571-2019 (PMC6853131; doi:10.5194/aab-62-571-2019)
Supplement: The supplement related to this article is available online at: https://doi.org/10.5194/aab-62-571-2019-supplement. [file aab-62-571-supplement.pdf]

Supplement of Arch. Anim. Breed., 62, 571–578, 2019
https://doi.org/10.5194/aab-62-571-2019-supplement
© Author(s) 2019. This work is distributed under
the Creative Commons Attribution 4.0 License.

Open Access
Archives Animal Breeding

Supplement of

Associations of *ORMDL1* gene copy number variations with growth traits in four Chinese sheep breeds

Xiaogang Wang et al.

Correspondence to: Hong Chen (chenhong1212@263.net) and Linyong Hu (xiangchou812@163.com)

The copyright of individual parts of the supplement might differ from the CC BY 4.0 License.

Table S1 Sex and age information of four Chinese sheep

**Breed**	**Sex**		**Age**			
	Male	Female	< 1 year old	1~2 years old	3~4 years old	5~6 years old
CK	191	109	0	220	62	18
HU	0	198	198	0	0	0
STH	93	89	0	182	0	0
LTH	17	37	0	54	0	0
